# Eight-year change in carotid intima-media thickness and associated risk factors in adults with and without psoriasis - the ELSA-Brasil cohort study

**DOI:** 10.1590/1414-431X2023e12609

**Published:** 2023-02-27

**Authors:** W.R. Tebar, I.S. Santos, V. Meneghini, M.S. Bittencourt, P.A. Lotufo, I.M. Bensenor

**Affiliations:** 1 Centro de Pesquisa Clínica e Epidemiológica, Hospital Universitário, Universidade de São Paulo, São Paulo, SP, Brasil; 2 Faculdade de Medicina, Universidade de São Paulo, São Paulo, SP, Brasil; 3 School of Medicine, University of Pittsburg, Pittsburg, USA

**Keywords:** Cardiovascular risk, Carotid intima-media thickness, Inflammatory disease, Psoriasis, Subclinical arterial disease

## Abstract

The longitudinal association between psoriasis and carotid intima-media thickness (CIMT) has not yet been established. This study aimed do compare CIMT and its change (∆CIMT) after an 8-year follow-up according to psoriasis diagnosis and the association with risk factors in the ELSA-Brasil study. Data from 7564 participants were analyzed (median age of 50.0 [44.0-57.0] years, 56.9% women). CIMT was assessed by ultrasound and ∆CIMT was calculated by subtracting baseline values from follow-up values. Psoriasis participants were identified by self-reported medical diagnosis (n=143) and compared with matched participants without disease (n=572) and with the entire sample without psoriasis (n=7421). Baseline CIMT explained the 8-year CIMT increase only in 36.9% among psoriasis participants and in ∼43.0% in participants without disease. CIMT was associated with age (β=0.002, P=0.002) and hypertension (β=0.029, P=0.034) in psoriasis participants. Among participants without psoriasis, CIMT was associated with age, male sex, low educational attainment, past smoking, obesity, diabetes, hypertension, and dyslipidemia (P<0.05). There was an inverse association of CIMT with private health insurance (β=-0.004, P=0.042) and White ethnicity (β=-0.006, P=0.004) in the entire sample without psoriasis but not in matched participants. Psoriasis participants showed an inverse association between ∆CIMT and diabetes (β=-0.214, P=0.011), while the entire sample without psoriasis showed an inverse association between ∆CIMT and age (β=-0.005, P<0.001), past smoking (β=-0.048, P=0.009), and hypertension (β=-0.048, P=0.009). In conclusion, psoriasis was not associated with CIMT after an 8-year follow-up. The inverse association of ∆CIMT with diabetes in psoriasis participants needs further clarification.

## Introduction

Psoriasis is a chronic inflammatory disease in which the immune system has a prominent role (1) and affects more than 125 million people in the world (2). Patients with psoriasis have a chronic state of systemic inflammation, higher oxidative stress processes, and dysfunctions in immune cells and adipose tissue, which may lead to an increased risk of atherosclerosis (3,4).

The carotid intima-media thickness (CIMT) is considered a surrogate marker of subclinical atherosclerosis (5) and a predictor of cardiovascular risk in patients with psoriasis (6). A meta-analysis reported that patients with psoriasis are associated with accelerated subclinical atherosclerosis, evidenced by greater CIMT compared with individuals without the disease (7). However, this evidence is refuted by some case-control studies (8) and remains controversial among population-based investigations, with positive (9) and negative (10) findings being reported. A narrative review of relevant PubMed results regarding association between CIMT and cardiovascular risk factors reported that both traditional and novel cardiovascular risk factors were associated with changes in CIMT, which include autoimmune diseases as psoriasis, but with inconsonant conclusions (11).

The progression of CIMT was associated with risk of subsequent cardiovascular events in the general population (12). The longitudinal association between CIMT increase and psoriasis is still scarcely studied. Analyzing whether CIMT increased more over time in patients with psoriasis than in patients without the disease is important for cardiovascular risk screening in this population. Thus, the present study aimed to prospectively analyze the CIMT of participants with and without the diagnosis of psoriasis in an 8-year follow-up using propensity score matching and considering the entire sample without psoriasis. The primary outcome was CIMT difference between groups according to diagnosis of psoriasis. The secondary outcome was the longitudinal association of CIMT with sociodemographic and traditional cardiovascular risk factors according to the diagnosis of psoriasis.

## Material and Methods

### Study design

This observational study analyzed longitudinal data from the Brazilian Longitudinal Study of Adult Health (ELSA-Brasil), a prospective cohort study that assessed Brazilian civil servants recruited from public educational and research institutions of six Brazilian state capitals (13). The present study used baseline data from the of ELSA-Brasil (2008-2010) and data from the third visit (2017-2019) collected eight years later. CIMT was measured at baseline and at the eight-year follow-up visit.

The data collection was conducted by an initial interview at the participant’s workplace and by a further visit to the Research Center for the clinical interviews and measurements. Annually, the researchers contacted the participants by phone to collect information about hospital and emergency admissions, medical consultation and procedures (surgeries and exams), and any possible changes in the participant’s health status. All these procedures were conducted by previously trained personnel under strict quality control, following standard protocols.

The ELSA-Brasil study was previously approved by the Ethical Research Committees from all the six institutions involved. All the research procedures were conducted according to the Declaration of Helsinki and all the participants signed an informed consent form.

### Diagnosis of psoriasis

Cases of psoriasis were identified according to medical diagnosis based on the participant’s report at the 8-year follow-up data collection visit (14), through the question: “Have you ever been diagnosed with psoriasis by a physician?” The positive respondents were asked to answer the second question: “What was your age at the first diagnosis of psoriasis?” This information was used to calculate disease duration by subtracting the age at diagnosis from the age of the participant at study enrollment. Spontaneous self-reported information of medical diagnosis of psoriasis was also considered for identification at baseline. The use of specific medications for the treatment of psoriasis was investigated among those who reported having the disease, being classified as “treated” (if any medication was reported) and “untreated” (without medication).

### Participant matching

**Figure 1:**
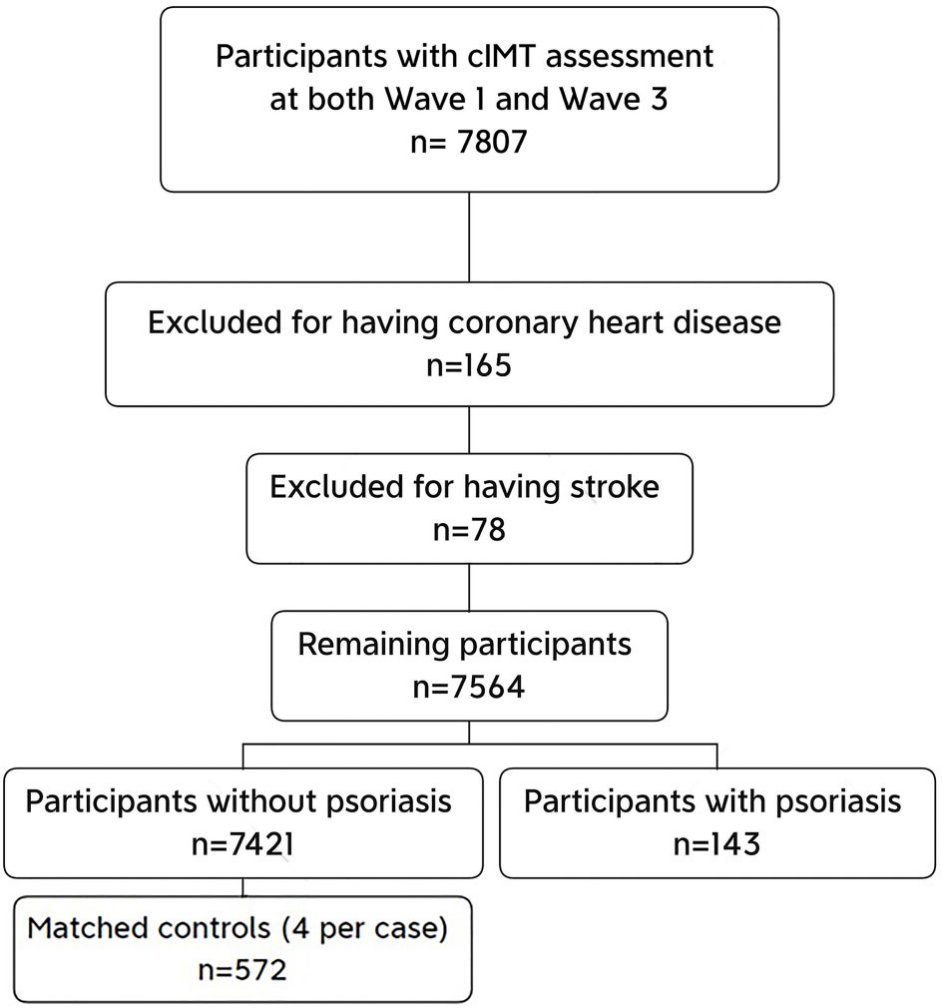
Flowchart of the sampling process. CIMT: Carotid intima-media thickness.

Participants from ELSA-Brasil with CIMT assessment at visits 1 and 3 were enrolled in this study (n=7,807). Data from 7,564 participants were included, after 165 exclusions due to prevalent coronary heart disease (heart attack or revascularization) and 78 exclusions due to prevalent stroke, both at baseline. At first, participants with diagnosis of psoriasis at baseline were identified (n=143). Four participants without psoriasis among the overall sample were paired for each participant with psoriasis by propensity score matching, considering age, sex, educational attainment, and having private health insurance. It was considered that pairing for cardiovascular risk factors would mitigate potential effects of psoriasis on CIMT, since this could be attributed to a different prevalence of these risk factors in participants with psoriasis compared to those without disease. Therefore, the group of matched participants without psoriasis totalized 572 participants (Figure 1).

### Carotid intima-media thickness (CIMT)

The carotid scans were obtained by a Toshiba Aplio XG ultrasound device (Japan) with a linear 7.5 Mhz transducer, being submitted to image quality assessment in the main reading center in São Paulo by trained technicians. Images were recorded during three cardiac cycles according to the American and Brazilian Societies of Echocardiography recommendations (15). The region of interest (ROI) for CIMT measurement in the ELSA-Brasil study was defined as a 1-cm long segment of the common carotid artery far wall, extending proximally from 1 cm below the carotid bulb. According to the protocol, this location was maintained irrespective of the presence of carotid plaques in the exam, aiming to minimize subjective and heterogeneous changes in the ROI, resulting in image reading changes. CIMT assessment was performed using the MIA software (USA). Board-certified technicians blinded for clinical information interpreted the data according to a standard protocol (15). MIA is a computer-aided program for a semi-automatic procedure: after the operator determines the ROI, no manual measurements are required. The software identifies the lumen-intima and media-adventitia interfaces, discretizes the ROI and obtains CIMT values for each frame during the video. CIMT values in a given exam are determined by the average of the measurements in each of its frames. The methodology of CIMT measurement in ELSA-Brasil has been previously published (16). The average values in millimeters (mm) from the measurements of left and right CIMT at baseline and 8-year follow-up were considered for analysis.

### Covariates

The variables of age, sex, educational attainment, ethnicity, private health insurance, obesity, smoking, alcohol consumption, physical activity, dyslipidemia, hypertension, and diabetes were considered as covariates.

Educational attainment was self-reported and the possible responses were: i) less than high school; ii) high school and some college; iii) at least college degree. Ethnicity was coded according to self-reported responses as used in the Brazilian census under the options: i) White; ii) Mixed; iii) Black; iv) Asian; and v) Indigenous. The responses were further classified as White and non-White. Physical activity was assessed by the International Physical Activity Questionnaire (IPAQ) validated for Brazilian adults (17), where participants were classified into physically active (meeting 150 min per week of combined moderate-to-vigorous exercise), or insufficiently active. When asked about smoking habit and alcohol consumption, participants answered whether they never, used to, or currently smoke/drink alcohol (13). Hypertension was defined as systolic blood pressure ≥140 mmHg or diastolic blood pressure ≥90 mmHg after three measurements with prior 5-min rest and 1-min interval between measurements during the visit, or taking antihypertensive medicines (18). The presence of diabetes was defined as medical history of diabetes, use of medication to treat diabetes, or fasting glycaemia ≥126 mg/dL (7.0 mmol/L) or oral glucose tolerance test ≥200 mg/dL (11.1 mmol/L), or glycated hemoglobin A1c ≥6.5% (48 mmol/mol) (19). Dyslipidemia was defined as medical history of dyslipidemia, use of lipid lowering medication, or low-density lipoprotein cholesterol ≥130 mg/dL after a 12-h fast (20). Obesity was defined as body mass index ≥30 kg/m^2^, which was calculated through objective anthropometric measurements, according to previously reported methods and instruments (13).

### Statistical analysis

Descriptive characteristics of the sample that were continuous variables are reported as median and interquartile range because of the non-normal distribution of data confirmed by Kolmogorov-Smirnov test, whereas categorical variables are reported as frequencies. Differences in proportions between groups were analyzed by the chi-squared test.

All the statistical analyses were performed in two steps: first, the variation in CIMT between visits 1 and 3 in participants with psoriasis was compared against the group of matched participants without psoriasis; secondly, the variation in CIMT between visits 1 and 3 in participants with psoriasis was compared against the overall sample without psoriasis.

Values of CIMT from baseline to follow-up were compared by the Wilcoxon test between participants with and without psoriasis. Delta values of CIMT (∆CIMT = follow-up - baseline values) according to psoriasis diagnosis were compared by the Mann-Whitney U test. Then, cross-sectional comparisons of CIMT (at baseline and at follow-up) and ∆CIMT were performed according to categories of independent variables. Values of CIMT and ∆CIMT were log10-transformed for data normalization in linear model analyses. Linear regression was performed to analyze the longitudinal relationship of independent variables with CIMT at follow-up and with ∆CIMT, where categorical independent variables were considered as factors and a reference category was defined for comparison of beta values. Repeated measures ANOVA was performed for CIMT (baseline *vs* follow-up) considering the psoriasis diagnosis as factor. Levene's test was used for equality of variances and resulted in P=0.459. Statistical significance was considered when P<0.05 using the SPSS Statistical Package version 25.0.

## Results

The 7564 included participants had a median age of 50.0 years (interquartile range 44.0 to 57.0), with no difference according to diagnosis of psoriasis (P=0.273). Of the participants with psoriasis (n=143), only 4.9% (n=7) reported taking medications to treat psoriasis, characterizing mild cases. Descriptive characteristics of the sample are presented in Table 1, considering participants with psoriasis *vs* matched participants without psoriasis and then considering participants with psoriasis *vs* the overall sample without psoriasis. Participants with psoriasis had higher prevalence of smoking than the matched control group (past smoking 33.6 *vs* 29.0% and current smoking 17.5 *vs* 10.3%, P=0.014) and higher proportion of private health insurance (74.1 *vs* 60.7%, P=0.001), higher educational attainment (complete college and more 65 *vs* 53.8%, P=0.024), and higher proportion of White ethnicity (67.1 *vs* 58.7%, P=0.024) than overall participants without psoriasis.

**Table 1:** Baseline characteristics of the sample.

	Participants with psoriasis (n=143)	Matched participants without psoriasis (n=572)	P-value (psoriasis *vs* matched participants)	Overall sample without psoriasis (n=7421)	P-value (psoriasis *vs* overall sample)
Age group			0.990		0.671
35-44	34 (23.8)	137 (24.0)		1960 (26.4)	
45-54	58 (40.6)	239 (41.8)		3093 (41.7)	
55-64	37 (25.9)	141 (24.7)		1815 (24.5)	
65-74	14 (9.8)	55 (9.6)		553 (7.5)	
Gender			0.762		0.808
Male	63 (44.1)	244 (42.7)		3194 (43.0)	
Female	80 (55.9)	328 (57.3)		4227 (57.0)	
Ethnicity			0.282		**0.024**
White	97 (67.1)	356 (62.2)		4355 (58.7)	
Mixed	31 (21.7)	121 (21.2)		1693 (22.8)	
Black	8 (5.6)	64 (11.2)		1007 (13.6)	
Asian	7 (4.9)	22 (3.8)		224 (3.0)	
Indigenous	0 (0.0)	3 (0.5)		65 (0.9)	
Not informed	1 (0.7)	6 (1.0)		77 (1.0)	
Educational attainment			0.501		**0.024**
Less than high school	9 (6.3)	23 (4.0)		769 (10.4)	
High school/incomplete college	41 (28.7)	169 (29.5)		2656 (35.8)	
Complete college or more	93 (65.0)	380 (66.4)		3996 (53.8)	
Private health insurance			0.800		**0.001**
No	37 (25.9)	154 (26.9)		2916 (39.3)	
Yes	106 (74.1)	418 (73.1)		4504 (60.7)	
Obesity			0.216		0.387
No	108 (75.5)	458 (80.2)		5826 (78.5)	
Yes	35 (24.5)	113 (19.8)		1593 (21.5)	
Smoking			**0.014**		0.062
Never	70 (49.0)	347 (60.7)		4328 (58.3)	
Past	48 (33.6)	166 (29.0)		2142 (28.9)	
Current	25 (17.5)	59 (10.3)		951 (12.8)	
Alcohol consumption			0.407		0.657
Never	15 (10.5)	41 (7.2)		750 (10.1)	
Past	23 (16.1)	91 (15.9)		1419 (19.1)	
Current	105 (73.4)	440 (76.9)		5252 (70.8)	
Physical activity			0.504		0.817
Inactive	85 (60.7)	330 (58.4)		4480 (61.6)	
Insufficiently active	20 (14.3)	68 (12.0)		911 (12.5)	
Active	35 (25.0)	167 (29.6)		1887 (25.9)	
Diabetes			0.232		0.453
No	119 (83.2)	498 (87.1)		6339 (85.5)	
Yes	24 (16.8)	74 (12.9)		1079 (14.5)	
Hypertension			0.873		0.836
No	100 (69.9)	403 (70.4)		5233 (70.5)	
Yes	43 (30.1)	169 (29.6)		2187 (29.5)	
Dyslipidemia			0.913		0.822
No	79 (56.0)	317 (55.5)		4218 (57.0)	
Yes	62 (44.0)	254 (44.5)		3185 (43.0)	

Data are reported as number (%). Bold type indicates statistical significance (chi-squared test).

**Figure 2:**
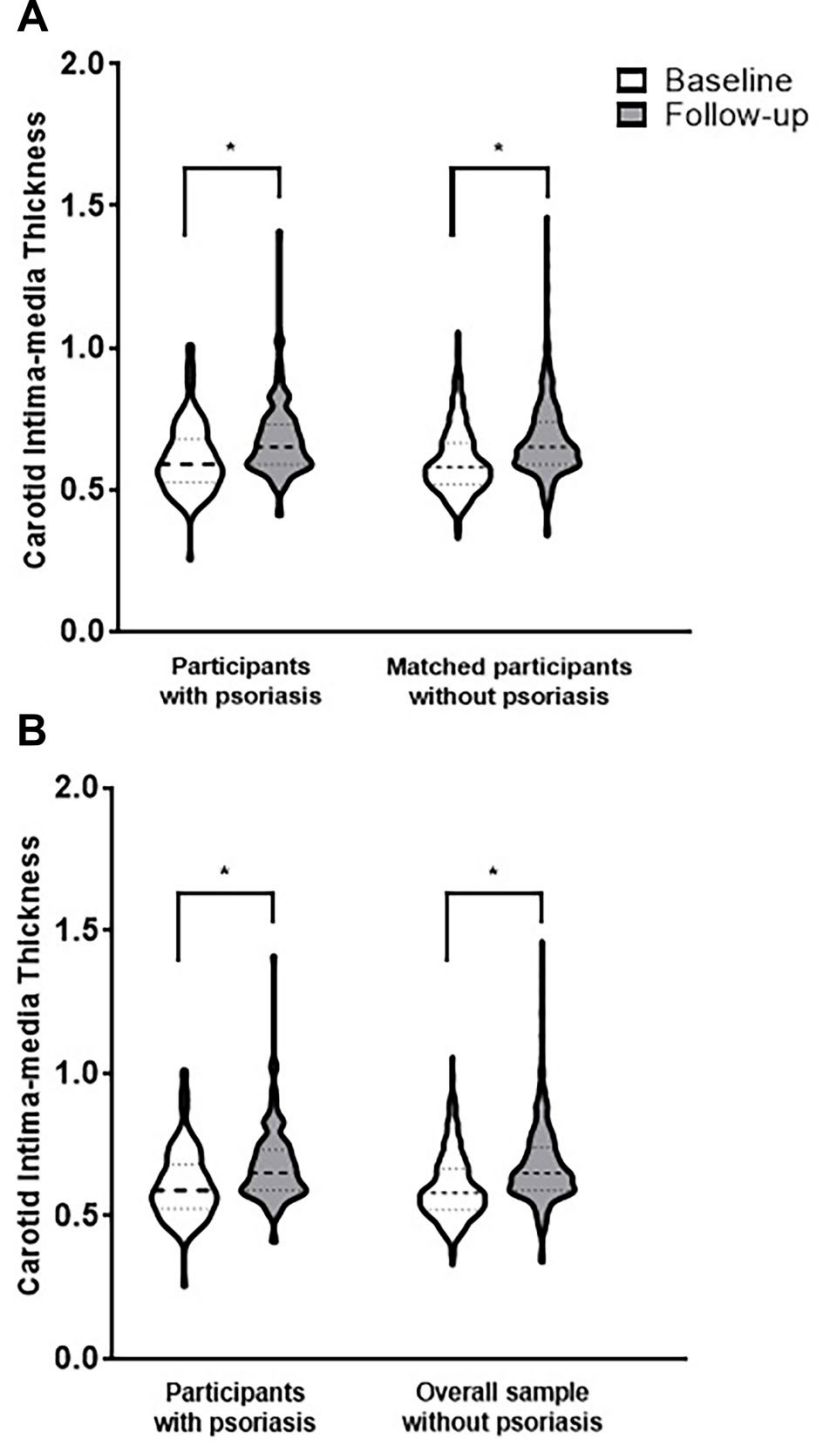
Violin plot of carotid intima-media thickness (in mm) at baseline and after the 8-year follow-up, according to diagnosis of psoriasis. *P<0.001 (Wilcoxon test).

CIMT significantly increased from baseline to follow-up in the sample. The median ∆CIMT values showed no significant difference between participants with psoriasis and matched controls (respective median values 0.070 mm [0.010; 0.110] *vs* 0.065 mm [0.020; 0.120]; Mann-Whitney U test P-value=0.553) and overall sample without psoriasis (respective median values 0.070 mm [0.010; 0.110] *vs* 0.070 mm [0.020; 0.125]; Mann-Whitney U test P-value=0.326). Participants with psoriasis showed an increase in median CIMT from 0.590 mm [0.525; 0.680] at baseline to 0.650 mm [0.585; 0.730] at follow-up (Wilcoxon test P-value <0.001). Matched controls showed an increase in median CIMT values from 0.580 mm [0.520; 0.665] to 0.645 mm [0.590; 0.735] (Wilcoxon test P-value <0.001), whereas overall sample without psoriasis showed an increase from 0.575 mm [0.510; 0.665] to 0.640 mm [0.585; 0.730] (Wilcoxon test P-value <0.001). No cross-sectional difference in median CIMT values was observed between participants with psoriasis and matched controls at baseline (P=0.372) and at follow-up (P=0.994) visits and overall sample without psoriasis at baseline (P=0.115) and at follow-up (P=0.689) moments. These results are reported in Figure 2.

The median values of CIMT and ∆CIMT according to categories of independent variables is presented in Supplementary Table S1. Participants with 60 years of age and more, who were obese, who were smokers, and those who had diabetes, hypertension, or dyslipidemia showed significantly lower ∆CIMT median values than their counterparts, and even had higher CIMT values at baseline. No difference in CIMT values was observed for alcohol consumption or physical activity. Difference in CIMT according private health insurance was observed only at follow-up, where participants who reported “Yes” had lower median CIMT value than those who reported “No”.

**Figure 3:**
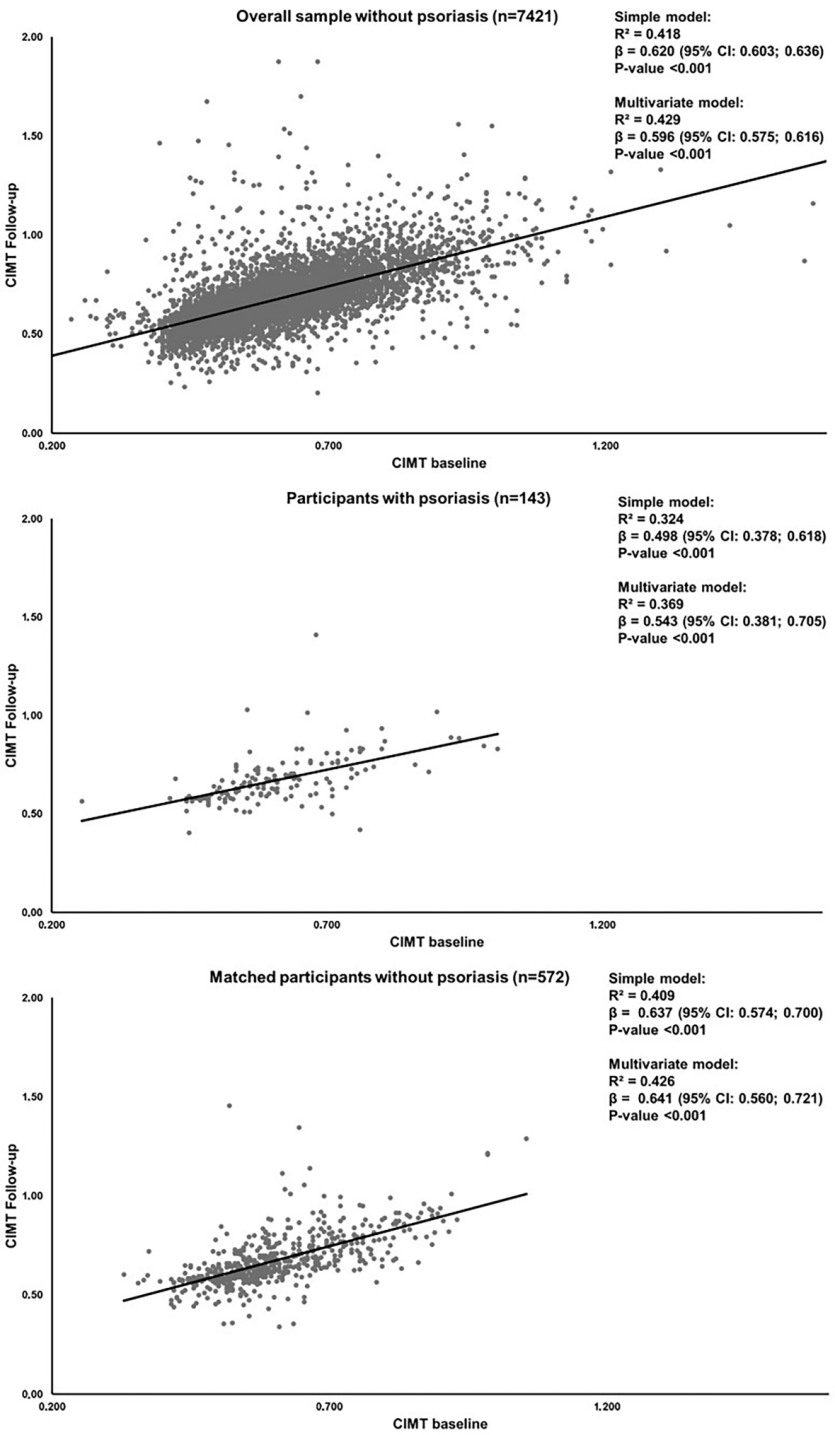
Scatterplot between baseline *vs* follow-up values of carotid intima-media thickness (CIMT), according to psoriasis diagnosis. β: angular coefficient of linear regression; CI: confidence interval. Multivariate model was simultaneously adjusted for age, sex, ethnicity, educational attainment, private health insurance, obesity, smoking, alcohol consumption, physical activity, diabetes, hypertension, and dyslipidemia.

The scatterplot of the relationship between CIMT values at baseline and at follow-up is presented in Figure 3. Participants with psoriasis showed lower angular coefficient (β=0.585, P<0.001) and R^2^ (0.313) compared to matched controls (β=0.741, P<0.001; R^2^=0.417) and compared to overall sample without psoriasis (β=0.702, P<0.001; R^2^=0.416). We also performed a multiple linear regression between baseline and follow-up CIMT adjusted by the interaction term psoriasis*CIMT at follow-up. In the analysis including participants with psoriasis and matched participants without psoriasis, the angular coefficient showed a slight decrease after adjustment for the interaction term (unadjusted β=0.706, P<0.001 *vs* adjusted β=0.694, P<0.001). When considering participants with psoriasis and the overall sample without psoriasis, the angular coefficient remained practically the same after adjustment for the interaction term (unadjusted β=0.699, P<0.001 *vs* adjusted β=0.698, P<0.001).

Table 2 presents the longitudinal relationship of CIMT at follow-up and ∆CIMT values with independent variables, according to the presence or absence of psoriasis. CIMT was associated with age in all three groups regardless of psoriasis. Among participants with psoriasis, CIMT was associated with age (P=0.002) and hypertension (P=0.034). Both groups without disease (matched participants and entire sample) showed a positive association of CIMT at follow-up with age, male sex, low educational attainment, past smoking, obesity, diabetes, hypertension, and dyslipidemia. In the entire sample without psoriasis, CIMT was also associated with current smoking and past alcohol consumption and inversely associated with private health insurance. Regarding ∆CIMT, psoriasis participants showed an inverse association with diabetes (P=0.011), while participants of the entire sample without psoriasis showed an inverse association of ∆CIMT with age, White ethnicity, past smoking, and hypertension. No associations with ∆CIMT were found for matched controls.

**Table 2:** Longitudinal associations between carotid intima-media thickness (CIMT) and sociodemographic and traditional cardiovascular risk factors in adults with and without psoriasis (PSO) (n=7564).

	CIMT at 8-year follow-up							
	PSO participants (n=143)		Matched participants (n=572)		Overall sample without PSO (n=7421)	
	β	P*-*value	β	P-value	β	P-value
Age	0.002	0.002*	0.003	<0.001*	0.003	<0.001*
Gender (men *vs* women as reference)	0.019	0.142	0.028	<0.001*	0.023	<0.001*
Ethnicity (White *vs* non-White as reference)	-0.013	0.329	-0.004	0.522	-0.006	0.004†
Educational attainment						
Complete college or more (reference)	-	-	-	-	-	-
High school or incomplete college	0.004	0.775	0.002	0.736	-0.001	0.491
Less than high school	0.017	0.512	0.047	0.006*	0.032	<0.001*
Private health insurance (“Yes” *vs* “No” as reference)	0.021	0.139	0.003	0.654	-0.004	0.042*
Smoking						
Never (reference)	-	-	-	-	-	-
Past	0.006	0.667	0.015	0.046†	0.019	<0.001*
Current	0.005	0.786	0.010	0.376	0.017	<0.001*
Alcohol consumption						
Never (reference)	-	-	-	-	-	-
Past	0.011	0.674	-0.002	0.896	0.010	0.007†
Current	0.008	0.685	-0.001	0.996	0.002	0.574
Physically active (“Yes” *vs* “No” as reference)	-0.011	0.464	0.007	0.309	-0.002	0.412
Obesity (“Yes” *vs* “No” as reference)	-0.003	0.825	0.024	0.004*	0.021	<0.001*
Diabetes (“Yes” *vs* “No” as reference)	-0.003	0.850	0.032	0.001†	0.036	<0.001*
Hypertension (“Yes” *vs* “No” as reference)	0.029	0.034†	0.035	<0.001*	0.037	<0.001*
Dyslipidemia (“Yes” *vs* “No” as reference)	0.001	0.945	0.015	0.027†	0.025	<0.001*

	∆CIMT					
	PSO participants (n=143)		Matched participants (n=572)		Overall sample without PSO (n=7421)	
	β	P-value	β	P-value	β	P-value
Age	-0.004	0.259	-0.007	0.101	-0.005	<0.001*
Gender (men *vs* women as reference)	0.001	0.993	-0.053	0.469	0.015	0.355
Ethnicity (White *vs* non-White as reference)	-0.038	0.523	-0.072	0.335	-0.034	0.039†
Educational attainment						
Complete college or more (reference)	-	-	-	-	-	-
High school or incomplete college	0.052	0.418	0.117	0.144	0.020	0.253
Less than high school	0.019	0.880	0.043	0.815	-0.016	0.571
Private health insurance (“Yes” *vs* “No” as reference)	0.000	0.998	-0.026	0.752	-0.014	0.403
Smoking						
Never (reference)	-	-	-	-	-	-
Past	0.046	0.479	-0.129	0.116	-0.048	0.009*
Current	0.040	0.617	0.008	0.949	0.017	0.502
Alcohol consumption						
Never (reference)	-	-	-	-	-	-
Past	-0.094	0.403	-0.020	0.897	-0.037	0.232
Current	-0.047	0.615	-0.099	0.470	-0.022	0.425
Physically active (“Yes” *vs* “No” as reference)	0.024	0.735	0.030	0.708	-0.018	0.324
Obesity (“Yes” *vs* “No” as reference)	0.013	0.853	0.139	0.128	0.027	0.169
Diabetes (“Yes” *vs* “No” as reference)	-0.214	0.011*	0.035	0.753	0.000	0.990
Hypertension (“Yes” *vs* “No” as reference)	-0.053	0.421	0.014	0.866	-0.048	0.009*
Dyslipidemia (“Yes” *vs* “No” as reference)	-0.091	0.115	0.046	0.532	-0.025	0.124

∆: follow-up minus baseline value. CIMT and ∆CIMT values were log10-transformed. *Remained significant after adjustment for all independent variables; †Lost significance after adjustment for all independent variables.

Table 3 presents the ANOVA results for CIMT at baseline and follow-up according to psoriasis. A significant difference in CIMT was found across time (from baseline to follow-up) in the analysis using matched participants without psoriasis and in the analysis including all participants without psoriasis. However, no effect was observed (psoriasis *vs* matched participants P-value=0.617; psoriasis *vs* overall sample P-value=0.377) and there was no significant interaction between psoriasis and time in both comparisons (psoriasis *vs* matched participants P-value=0.813; psoriasis *vs* overall sample P-value=0.424).

**Table 3:** Repeated measures ANOVA of carotid intima-media thickness at baseline and at the 8-year follow-up of ELSA-Brasil cohort participants, according to psoriasis diagnosis (n=7564).

	Participants with psoriasis (n=143)	Matched participants without psoriasis (n=572)	Effect P-value		
	Estimated mean (95%CI)	Estimated mean (95%CI)	Psoriasis	Time	Psoriasis*Time
CIMT			0.616	<0.001	0.377
Baseline	0.610 (0.591; 0.629)	0.600 (0.591; 0.610)			
Follow-up	0.674 (0.652; 0.695)	0.673 (0.662; 0.684)			
CIMT log10-transformed			0.593	<0.001	0.462
Baseline	-0.223 (-0.236; -0.210)	-0.229 (-0.236; -0.222)			
Follow-up	-0.178 (-0.191; -0.165)	-0.180 (-0.186; -0.173)			

	Participants with psoriasis (n=143)	Overall sample(n=7421)	Effect P-value		
	Estimated mean (95%CI)	Estimated mean (95%CI)	Psoriasis	Time	Psoriasis*Time
CIMT			0.466	<0.001	0.332
Baseline	0.610 (0.589; 0.631)	0.598 (0.595; 0.601)			
Follow-up	0.674 (0.651; 0.696)	0.671 (0.668; 0.674)			
CIMT log10-transformed			0.337	<0.001	0.331
Baseline	-0.223 (-0.237; -0.209)	-0.232 (-0.234; -0.230)			
Follow-up	-0.178 (-0.192; -0.165)	-0.182 (-0.184; -0.180)			

CIMT: carotid intima-media thickness; CI: confidence interval.

## Discussion

The present study observed that psoriasis was not associated with CIMT among ELSA-Brasil participants after an 8-year follow-up, whereas ∆CIMT was inversely associated with different cardiovascular risk factors in participants with and without psoriasis.

Santos et al. (16) reported that more than 60% of CIMT variability is not explained by sociodemographic and traditional risk factors and this number may even be lower in the Northern Manhattan Study (21). Since psoriasis was not associated with higher CIMT compared to individuals without psoriasis in the present study, it is not possible to infer about the role of this specific disease as a novel determinant of changes in CIMT over time. This may be at least partially due to the low number of participants with psoriasis in the sample (n=143). In addition, the higher prevalence of smoking among psoriasis participants corroborates with previous findings (22). A possible dose-effect of smoking intensity and duration with psoriasis incidence (23) and an impact on severity of disease and response to treatment have been reported (24). Overall, the ELSA-Brasil cohort presented a low prevalence of smoking compared to the global smoking prevalence of 32.6% among men and 6.5% among women (25).

CIMT after the 8-year follow-up was associated with age and hypertension in participants with psoriasis. The increase in CIMT over time was an expected outcome due to the significant association between CIMT and aging, as reported by previous studies (26). The longitudinal relationship between CIMT and hypertension in the present study corroborated with previous findings in the literature (27). Participants with psoriasis howed an inverse association of ∆CIMT with diabetes, while in the overall sample without psoriasis, an inverse association with hypertension was found, which were unexpected results, since diabetes and hypertension are risk factors for carotid atherosclerosis (28,29). In a previous population-based cohort study, van den Berg et al. (30) observed no vascular risk factors associated with change in CIMT over a 5-year follow-up. The presence of a ceiling effect on ∆CIMT regardless of the presence of psoriasis needs to be further investigated to determine its possible influence on the findings of this study, since participants with traditional risk factors presented lower ∆CIMT values despite having higher CIMT values at both baseline and follow-up.

Another possible explanation for the inverse association of ∆CIMT with diabetes and hypertension is the use of medications, since statin use was higher among participants with hypertension (20.6 *vs* 7.2% in those without hypertension, P<0.001) and diabetes (25.0 *vs* 8.8% in those without diabetes, P<0.001), but was not significantly different between participants with and without psoriasis (13.7 *vs* 11.1%, P=0.338). A meta-analysis reported that the use of atorvastatin was associated with a significant reduction in CIMT among participants with type 2 diabetes (31) and the use of simvastatin associated with antihypertensive medication significantly decreased CIMT (32). The use of low-dose metoprolol CR/XL beta-blocker and fluvastatin was shown to reduce the rate of progression of CIMT in clinically healthy subjects with carotid plaque (33). Mörtsell et al. (34) observed a reduction in CIMT after 48 weeks of use of irbesartan, whereas the group treated with atenolol showed an increase in CIMT. However, one study reported no effect of two-year statin therapy on CIMT, but a lower rate of cardiovascular events in these patients (35). In addition, participants of the ELSA-Brasil cohort had better access to medical treatment compared to the Brazilian population, and the frequency of using medication to treat hypertension, diabetes, and especially dyslipidemia increased from baseline to 8 years of follow-up.

Regarding the inverse association of ∆CIMT with age in the overall sample without psoriasis observed in the present study, a two-sided hypothesis may be speculated: i) younger participants are more likely to have lower CIMT and consequently greater risk of CIMT increase over time than those who already have higher CIMT; and ii) on the other hand, elderly participants with higher ∆CIMT were more likely to die or less likely to participate in the follow-up visit. The second hypothesis could indicate a survivorship bias in the present study, which needs to be further investigated by fatal/non-fatal cardiovascular events analysis during the study follow-up.

An inverse association of ∆CIMT with White ethnicity was found for the entire sample without psoriasis. In general, Black people in Brazil have lower educational attainment, lower income, and less access to health services, resulting in a poorer health condition. The inverse association between ∆CIMT and past smoking in the present study could be related to the higher risk of atherosclerosis among smokers, as smoking cessation could have been motivated by previous health problems in a group with an already high CIMT, resulting in a lower increase over the time.

Although the present study found no difference in ∆CIMT after the 8-year follow-up according to psoriasis diagnosis, information about longitudinal changes in participants with and without psoriasis was not found in the literature. Previous cross-sectional studies reported associations between psoriasis and increased CIMT (6,9,36), but this evidence is not consensual (8,10). Some methodological aspects may justify the lack of consensus, such as sample recruitment (dermatology clinics *vs* population-based) and comparator groups (convenience healthy matched controls, nested case-controls, general sample comparisons).

Besides study methodologies, different characteristics of the participants with psoriasis could compromise convergence of findings, such as the age range, type of treatment, and severity of disease. Martinez-Lopez et al. (37) observed that participants with moderate and severe psoriasis treated with immunobiological drugs (mainly anti-IL-12/23) presented a decrease in CIMT levels after an 8-month follow-up compared to patients treated with systemic drugs (mainly methotrexate). Bańska-Kisiel et al. (38) reported that severity of disease was associated with CIMT even in participants with mild and moderate psoriasis. In our sample, an occupational cohort study with 80% of active workers, most cases were mild because few participants were under treatment (4.9%) and use of immunobiological drugs was not reported in patients with psoriasis although it was reported for other diseases.

Although participants with psoriasis have been found to have a higher risk of atherosclerosis (3,31), the present study observed that ∆CIMT was not different in participants with psoriasis compared with matched participants and with the overall sample. Pezzolo et al. (39) reported a bimodal distribution pattern of psoriasis incidence, with peaks at 35-44 years of age (early-onset) and between 65-74 years of age (late-onset). In this sense, it is possible that ELSA-Brasil participants, most of whom are employed, are less likely to have severe disease, since only 7.5% of the participants in the present study were in the late-onset psoriasis age group (65-74 years) and less than 10% of the participants with psoriasis diagnosis were in this age range (n=14), so this group may be underrepresented in our sample and participants of this age could be more likely to have atherosclerosis than younger participants with psoriasis.

It is important to highlight the limitations of the present study. First, the small sample of participants with psoriasis consisted of mild cases with low frequency of treatment, which precluded the investigation of treatment status affecting the results and disease severity. Second, in a highly mixed sample such as that of ELSA-Brasil, with a high proportion of non-white participants and a proportion of active workers of approximately 80%, could be biased by the “healthy worker bias”, whereas a “survival bias” cannot be excluded either. Third, this study did not analyze the presence and extent of carotid plaques, which may be more representative of atherosclerosis than CIMT (40).

On the other hand, the CIMT assessment followed a strict protocol with image processing performed in a unique reading center by computerized edge-tracking with multiframe method to ensure correct IMT measurements. In addition, the eight-year follow-up, the statistical analysis adjusted by potential confounding factors, and the comparison group with four controls per case paired by sex, age, educational attainment, and private health insurance could be considered strengths of this study. Since the association of psoriasis with subclinical cardiovascular disease is still controversial, with both positive and negative results, the present longitudinal investigation provides novel information about a highly mixed sample on the breadth of literature.

In conclusion, CIMT was significantly increased after eight-years of follow-up in both participants with and without psoriasis. The baseline and follow-up CIMT values, as well as ∆CIMT did not differ according to psoriasis diagnosis in the ELSA-Brasil cohort study. The determinants of the inverse association between ∆CIMT and age, diabetes, and hypertension in this cohort needs to be further investigated to clarify the hypothesis raised by this study of survival bias and high access to medical treatment. Other studies analyzing ∆CIMT in different populations are also needed to compare these findings. A longer-term follow-up through future waves of data collection from ELSA-Brasil should provide more robust evidence and well-detectable CIMT changes in participants with psoriasis.

## Supplementary Material

Click to view [pdf].

## References

[1] Lebwohl M (2003). Psoriasis. Lancet.

[2] Greb JE, Goldminz AM, Elder JT, Lebwohl MG, Gladman BB, Wu JJ (2016). Psoriasis. Nat Rev Dis Primers.

[3] Sajja AP, Joshi AA, Teague HL, Dey AK, Mehta NN (2018). Potential immunological links between psoriasis and cardiovascular disease. Front Immunol.

[4] Garshick MS, Barrett TJ, Wechter T, Azarchi S, Scher JU, Neimann A (2019). Inflammasome signaling and impaired vascular health in psoriasis. Arterioscler Thromb Vasc Biol.

[5] Bauer M, Caviezel S, Teynor A, Erbel R, Mahabadi AA, Schmidt-Trucksäss A (2012). Carotid intima-media thickness as a biomarker of subclinical atherosclerosis. Swiss Med Wkly.

[6] Girisha BS, Shibina S, Raghuraja U, Subramanyam K (2021). Carotid intima-media thickness and epicardial fat thickness predict precoronary artery disease status in psoriasis. Indian J Dermatol Venereol Leprol.

[7] Fang N, Han W, Gong D, Zou Chen, Fan Y (2015). Atorvastatin treatment for carotid intima-media thickness in Chinese patients with type 2 diabetes: a meta-analysis. Medicine (Baltimore).

[8] Kim SY, Yang HS, Lee YW, Choe YB, Ahn KJ (2015). evaluation of the beta stiffness index and carotid intima-media thickness in Asian patients with psoriasis. Angiology.

[9] Troitzsch P, Markus MRP, Dörr M, Felix SB, Jünger M, Schminke U (2012). Psoriasis is associated with increased intima-media thickness--the Study of Health in Pomerania (SHIP). Atherosclerosis.

[10] Dowlatshahi EA, Kavousi M, Nijsten T, Ikram MA, Hofman A, Franco OH (2013). Psoriasis is not associated with atherosclerosis and incident cardiovascular events: the Rotterdam study. J Invest Dermatol.

[11] Qu B, Qu T (2015). Causes of changes in carotid intima-media thickness: a literature review. Cardiovasc Ultrasound.

[12] Lorenz MW, Polak JF, Kavousi M, Mathiesen EB, Völzke H, Tuomainen TP (2012). Carotid intima-media thickness progression to predict cardiovascular events in the general population (the PROG-IMT collaborative project): a meta-analysis of individual participant data. Lancet.

[13] Aquino EM, Barreto SM, Benseãor IM, Carvalho MS, Chor D, Duncan BB (2012). Brazilian Longitudinal Study of Adult Health (ELSA-Brasil): objectives and design. Am J Epidemiol.

[14] Bensenor IM, Goulart AC, Pereira AC, Brunoni AR, Alencar A, Santos RD (2022). Chronic inflammatory diseases, subclinical atherosclerosis, and cardiovascular diseases: design, objectives, and baseline characteristics of a prospective case-cohort study - ELSA-Brasil. Clinics (Sao Paulo).

[15] Mill JG, Pinto K, Griep RH, Goulart A, Foppa M, Lotufo PA (2013). Medical assessments and measurements in ELSA-Brasil [in Portuguese]. Rev Saude Publica.

[16] Santos IS, Alencar AP, Rundek T (2015). Low Impact of Traditional Risk Factors on Carotid Intima-Media Thickness: The ELSA-Brasil Cohort. Arterioscler Thromb Vasc Biol.

[17] Hallal PC, Victora CG (2004). Reliability and validity of the International Physical Activity Questionnaire (IPAQ). Med Sci Sports Exerc.

[18] Nascimento LR, Molina MC, Faria CP, Cunha RS, Mill JG (2013). Reproducibility of arterial pressure measured in the ELSA‐Brasil with 24‐hour pressure monitoring [in Portuguese]. Rev Saude Publica.

[19] Schmidt MI, Hoffmann JF, Diniz MFS, Lotufo PA, Griep RH, Bensenor IM (2014). High prevalence of diabetes and intermediate hyperglycemia - The Brazilian Longitudinal Study of Adult Health (ELSA-Brasil). Diabetol Metab Syndr.

[20] Santos RD, Bensenor IM, Pereira AC, Lotufo PA (2016). Dyslipidemia according to gender and race: the Brazilian Longitudinal Study of Adult Health (ELSA-Brasil). J Clin Lipidol.

[21] Rundek T, Blanton SH, Bartels S, Dong C, Raval A, Demmer RT (2013). Traditional risk factors are not major contributors to the variance in carotid intima-media thickness. Stroke.

[22] Gazel U, Ayan G, Solmaz D, Akar S, Aydin SZ (2020). The impact of smoking on prevalence of psoriasis and psoriatic arthritis. Rheumatology (Oxford).

[23] Armstrong AW, Harskamp CT, Dhillon JS, Armstrong EJ (2014). Psoriasis and smoking: a systematic review and meta-analysis. Br J Dermatol.

[24] Naldi L (2016). Psoriasis and smoking: links and risks. Psoriasis (Auckl).

[25] Dai X, Gakidou E, Lopez AD (2022). Evolution of the global smoking epidemic over the past half century: strengthening the evidence base for policy action. Tob Control.

[26] Madhuri V, Chandra S, Jabbar A (2010). Age associated increase in intima media thickness in adults. Indian J Physiol Pharmacol.

[27] Zhang L, Fan F, Qi L, Jia J, Yang Y, Li J (2019). The association between carotid intima-media thickness and new-onset hypertension in a Chinese community-based population. BMC Cardiovasc Disord.

[28] Wagenknecht LE, Zaccaro D, Espeland MA, Karter AJ, O'Leary DH, Haffner SM (2003). Diabetes and progression of carotid atherosclerosis: the insulin resistance atherosclerosis study. Arterioscler Thromb Vasc Biol.

[29] Su TC, Jeng JS, Chien KL, Sung FC, Hsu HC, Lee YT (2001). Hypertension status is the major determinant of carotid atherosclerosis: a community-based study in Taiwan. Stroke.

[30] van den Berg E, Biessels GJ, Stehouwer CD, Kappelle LJ, Heine RJ, Nijpels G (2010). Ten-year time course of risk factors for increased carotid intima-media thickness: the Hoorn Study. Eur J Cardiovasc Prev Rehabil.

[31] Fang N, Jiang M, Fan Y (2016). Association between psoriasis and subclinical atherosclerosis: a meta-analysis. Medicine (Baltimore).

[32] de Sauvage Nolting PR, de Groot E, Zwinderman AH, Buirma RJ, Trip MD, Kastelein JJ (2003). Regression of carotid and femoral artery intima-media thickness in familial hypercholesterolemia: treatment with simvastatin. Arch Intern Med.

[33] Hedblad B, Wikstrand J, Janzon L, Wedel H, Berglund G (2001). Low-dose metoprolol CR/XL and fluvastatin slow progression of carotid intima-media thickness: Main results from the Beta-Blocker Cholesterol-Lowering Asymptomatic Plaque Study (BCAPS). Circulation.

[34] Mörtsell D, Malmqvist K, Held C, Kahan T (2007). Irbesartan reduces common carotid artery intima-media thickness in hypertensive patients when compared with atenolol: the Swedish Irbesartan Left Ventricular Hypertrophy Investigation versus Atenolol (SILVHIA) study. J Intern Med.

[35] Beishuizen ED, van de Ree MA, Jukema JW, Tamsma JT, van der Vijver JCM, Meinders AE (2004). Two-year statin therapy does not alter the progression of intima-media thickness in patients with type 2 diabetes without manifest cardiovascular disease. Diabetes Care.

[36] Haberka M, Bańska-Kisiel K, Bergler-Czop B (2018). Mild to moderate psoriasis is associated with oxidative stress, subclinical atherosclerosis, and endothelial dysfunction. Pol Arch Intern Med.

[37] Martinez-Lopez A, Blasco-Morente G, Perez-Lopez I, Tercedor-Sanchez J, Arias-Santiago S (2018). Studying the effect of systemic and biological drugs on intima-media thickness in patients suffering from moderate and severe psoriasis. J Eur Acad Dermatol Venereol.

[38] Bańska-Kisiel K, Haberka M, Bergler-Czop B, Brzezińska-Wcisło L, Okopień B, Gąsior Z (2016). Carotid intima-media thickness in patients with mild or moderate psoriasis. Postepy Dermatol Alergol.

[39] Pezzolo E, Cazzaniga S, Colombo P, Chatenoud L, Naldi L (2019). Psoriasis incidence and lifetime prevalence: suggestion for a higher mortality rate in older age-classes among psoriatic patients compared to the general population in Italy. Acta Derm Venereol.

[40] Spence JD (2012). Carotid plaque measurement is superior to IMT Invited editorial comment on: carotid plaque, compared with carotid intima-media thickness, more accurately predicts coronary artery disease events: a meta-analysis-Yoichi Inaba, M.D., Jennifer A. Chen M.D., Steven R. Bergmann M.D., Ph.D. Atherosclerosis.

